# Intravenous Administration of Ad26.COV2.S Does Not Induce Thrombocytopenia or Thrombotic Events or Affect SARS-CoV-2 Spike Protein Bioavailability in Blood Compared with Intramuscular Vaccination in Rabbits

**DOI:** 10.3390/vaccines11121792

**Published:** 2023-11-30

**Authors:** Selina Khan, Sonia Marquez-Martinez, Tim Erkens, Adriaan de Wilde, Lea M. M. Costes, Petra Vinken, Sandra De Jonghe, Wendy Roosen, Chiara Talia, Ronnie Chamanza, Jan Serroyen, Jeroen Tolboom, Roland C. Zahn, Frank Wegmann

**Affiliations:** 1 Janssen Vaccines & Prevention, 2333 CN Leiden, The Netherlands; smarque9@its.jnj.com (S.M.-M.); a.h.dewilde@gmail.com (A.d.W.); l.m.m.costes@gmail.com (L.M.M.C.); jserroy1@its.jnj.com (J.S.); jtolboom@its.jnj.com (J.T.); fwegmann@its.jnj.com (F.W.); 2 Janssen Research & Development—A Division of Janssen Pharmaceutica NV, 2340 Beerse, Belgium; terkens1@its.jnj.com (T.E.); sdjonghe@its.jnj.com (S.D.J.); wendyroosen@outlook.com (W.R.); ctalia@its.jnj.com (C.T.);

**Keywords:** adenovirus, viral vector, vaccine, platelets, rabbits

## Abstract

Vaccine-induced immune thrombotic thrombocytopenia (VITT) is a very rare but serious adverse reaction that can occur after Ad26.COV2.S vaccination in humans, leading to thrombosis at unusual anatomic sites. One hypothesis is that accidental intravenous (IV) administration of Ad26.COV2.S or drainage of the vaccine from the muscle into the circulatory system may result in interaction of the vaccine with blood factors associated with platelet activation, leading to VITT. Here, we demonstrate that, similar to intramuscular (IM) administration of Ad26.COV2.S in rabbits, IV dosing was well tolerated, with no significant differences between dosing routes for the assessed hematologic, coagulation time, innate immune, or clinical chemistry parameters and no histopathologic indication of thrombotic events. For both routes, all other non-adverse findings observed were consistent with a normal vaccine response and comparable to those observed for unrelated or other Ad26-based control vaccines. However, Ad26.COV2.S induced significantly higher levels of C-reactive protein on day 1 after IM vaccination compared with an Ad26-based control vaccine encoding a different transgene, suggesting an inflammatory effect of the vaccine-encoded spike protein. Although based on a limited number of animals, these data indicate that an accidental IV injection of Ad26.COV2.S may not represent an increased risk for VITT.

## 1. Introduction

Ad26.COV2.S is a monovalent vaccine composed of a recombinant, replication-incompetent human adenovirus type 26 (Ad26) vector, encoding a full-length, membrane-bound severe acute respiratory syndrome coronavirus-2 (SARS-CoV-2) spike (S) protein (Wuhan strain) stabilized in its prefusion conformation [1].

Ad26.COV2.S has been used in millions of individuals for the prevention of COVID-19 and is highly effective against severe and critical COVID-19, COVID-19-related hospitalization, and death, with protection lasting ≥6 months [2].

Thrombosis with thrombocytopenia syndrome (TTS) has been reported following vaccination with ChAdOx1 nCoV-19, Ad26.COV2.S [3,4,5,6], and, to a lesser extent, with COVID-19 mRNA-1273 [7], inactivated COVID-19 vaccines [8,9], and Gam-COVID-Vac [10]; some similar cases were observed following COVID-19 disease [11,12]. TTS has occurred as a very rare event in approximately 2.3 to 5.5 cases per 1 million doses of Ad26.COV2.S administered, depending on the definition of TTS (Centers for Disease Control [USA] and Prevention of Pharmacovigilance Risk Assessment Committee [EMA]) [5,13,14]. The very low incidence rate complicates the identification of a causal pathway leading to this adverse clinical outcome. The term vaccine-induced immune thrombotic thrombocytopenia (VITT) has been used to describe cases that are likely vaccine adverse effect-related.

The hallmarks of VITT are thrombosis at unusual anatomic sites, such as brain venous sinuses or the splanchnic vein, severe thrombocytopenia with the presence of platelet-activating antibodies targeting platelet factor 4 (PF4), and high D-dimer levels [15]. The onset of symptoms usually occurs within 5 to 43 days following vaccination and can be fatal; however, with appropriate treatment, the symptoms can be managed [3,4,5,6,16].

The mechanistic relevance of PF4 antibodies was shown recently by demonstrating PF4-VITT antibody complex-induced thrombus formation in vitro, which was mediated through neutrophilic activation and NETosis in a FcγRIIa-dependent manner. The adoptive transfer of purified VITT IgG into a human PF4 and FcγRIIa transgenic mouse model confirmed these data [16,17]. Furthermore, anti-PF4 antibodies from patients with VITT bind to a highly restricted epitope site on PF4 that corresponds to the heparin-binding site and are reported to have the same single lambda light chain IGLV3-21 [18], and therefore have a distinct specificity compared with antibodies found in heparin-induced thrombocytopenia [17,19].

Multiple hypotheses have been proposed for potential mechanisms underlying VITT post-vaccination with adenovirus vector-based COVID-19 vaccines. One hypothesis is that systemic exposure of adenovirus vector-based vaccine particles and/or the S protein encoded by the vector-based vaccines (due to either accidental intravenous [IV] injection or leakage from the muscle injection site into the blood) may lead to interaction of the adenovector with platelets [3,20,21,22]. Nicolai and colleagues reported that IV, but not intramuscular (IM), injection of a high dose of ChAdOx1 nCoV-19 in mice resulted in platelet-adenovirus aggregate formation and platelet activation [20,22]. A possible contributing factor to this effect may be IV dosing-related systemic biodistribution of the vaccine-encoded S protein, which has been shown to activate platelets and cause thrombus formation and inflammatory responses in mice [20,23].

Here, we investigated whether IV dosing (as a model for accidental systemic exposure) of Ad26.COV2.S could induce signs of thromboembolic disease or changes in platelet counts, other clinical pathology parameters, histopathology findings (of thrombotic events and their sequelae), or systemic S protein exposure when compared with IM dosing in rabbits.

## 2. Materials and Methods

### 2.1. Ethics Statement

The rabbit study was conducted at Janssen Research and Development Belgium in facilities approved by the Institute of Health Office of Animal Welfare and accredited by the Association for Assessment and Accreditation of Laboratory Animal Care.

Animal research protocols were approved by the Institutional Ethical Committee, and the studies were conducted in compliance with the European Convention for the Protection of Vertebrate Animals Used for Experimental and Other Scientific Purposes and Belgian guidelines, with the principles of euthanasia as stated in the guidelines from the American Veterinary Medical Association Panel [24]. Import and export permits for vectors and rabbit biospecimens were obtained in compliance with European Union federal regulations.

The mouse study was performed at Janssen Vaccines and Prevention, The Netherlands, and was conducted according to the Dutch Animal Experimentation Act and the Guidelines on the Protection of Animals for Scientific Purposes by the Council of the European Committee after approval by the Centrale Commissie Dierproeven and the Dier Experimenten Commissie.

### 2.2. Vaccines

Replication-incompetent, E1/E3-deleted recombinant Ad26 vectors were engineered using the AdVac^®^ system, as described elsewhere [25,26], with Ad26 encoding stabilized SARS-CoV-2 S protein (Ad26.COV2.S) [1] or the envelope (Env) protein of Zika virus (Ad26.ZIKV.001) [26,27]. The vectors were clinical grade material produced under the same process.

Ad26-mediated expression of the various transgenes was confirmed by Western blot analysis of cell-culture lysates from infected A549 cells or through a polymerase chain reaction (PCR).

The commercial clinical grade measles–mumps–rubella (MMR) vaccine (M-M-RVAXPRO, Merck Sharp and Dohme BV, Haarlem, Netherlands) was used as a control in the rabbit study.

### 2.3. Animals and Housing

A total of 45 eleven-week-old healthy female New Zealand white rabbits (body weight [BW] 2.1–2.8 kg at study start) were included. Female rabbits were used since, at the start of the rabbit study, TTS cases observed appeared primarily in female patients; however, this imbalance is presently less clear. The animals were supplied by Charles River Laboratories (France). Rabbits were kept in a biosafety level 2 facility under specific pathogen-free conditions after screening negative for Mycobacterium tuberculosis, simian immunodeficiency virus, simian retrovirus, and simian T-lymphotropic virus. Screening included herpes B virus and measles serology. Rabbits were single-housed in stainless steel cages with a slotted plastic floor and placed in study-dedicated rooms.

For our study in mice, six- to eight-week-old naïve specific-pathogen-free female BALB/c mice (Charles River Laboratories) were housed with 10 animals per cage.

For all animal studies, animals were kept under controlled, recorded environmental conditions of humidity and temperature and a 12 h light cycle. Animals were provided with sensory and cognitive environmental enrichment. Animals were fed a standard diet ad libitum, and tap water was provided ad libitum through an automated system. Animal well-being and health surveillance was monitored at least twice daily by husbandry staff. Preset humane end points were used by a veterinarian to define sacrifice criteria not related to the study. All measures were taken to minimize pain, distress, and suffering, and all procedures were performed by trained personnel.

### 2.4. Study Design and Animal Procedures

**Figure A1:**
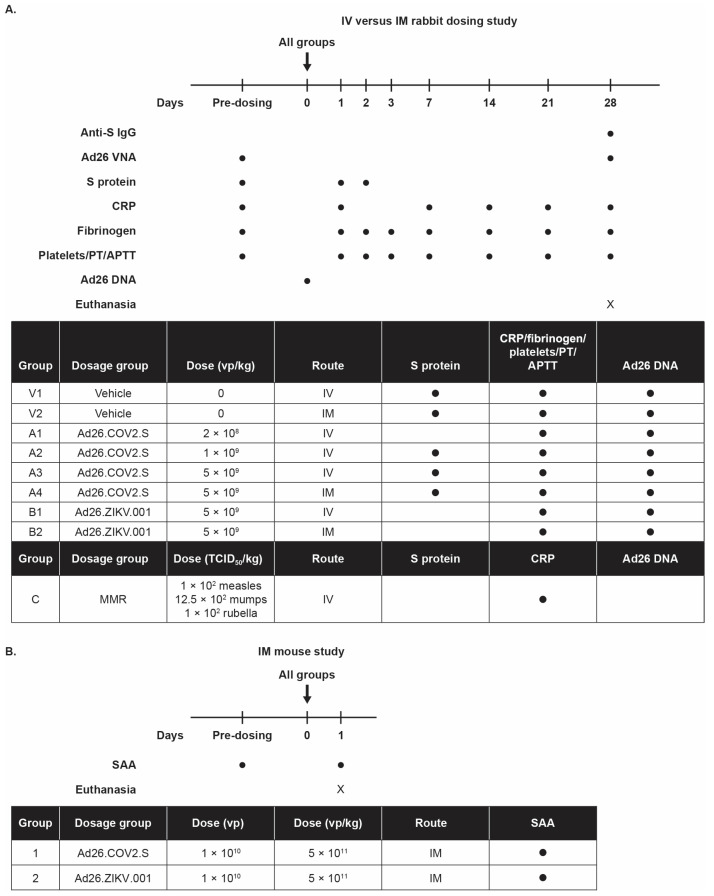
Study design. (**A**) Rabbits received Ad26.COV2.S, Ad26.ZIKV.001, MMR vaccines, or vehicle at the indicated dose levels either IV or IM. N = 8 per group. Serum and plasma were collected at the indicated time points and processed for the assays listed. Rabbits were euthanized on day 28 post-dosing. (**B**) Mice were IM dosed with 10^10^ vp of either Ad26.COV2.S or Ad26.ZIKV.001, sera were collected 24 h later, and mice were then euthanized. APTT, activated partial thromboplastin time; CRP, C-reactive protein; IM, intramuscular; IV, intravenous; MMR, measles–mumps–rubella; PT, prothrombin time; S, spike; SAA, serum amyloid A; TCID_50_, 50% tissue culture infectious dose; VNA, virus neutralization assay; vp, viral particles.

Rabbits were divided into nine study groups with five animals per group (Figure A1A). The animals received a single dose of Ad26.COV2.S IV (0.2 × 10^9^ viral particles [vp]/kg, 1 × 10^9^ vp/kg, or 5 × 10^9^ vp/kg), Ad26.COV2.S IM (5 × 10^9^ vp/kg), Ad26.ZIKV.001 IV (5 × 10^9^ vp/kg), Ad26.ZIKV.001 IM (5 × 10^9^ vp/kg), or a full human dose (FHD) of MMR vaccine (10^2^ measles 50% tissue culture infectious dose [TCID_50_]/kg, 12 × 10^2^ mumps TCID_50_/kg, 10^2^ rubella TCID_50_/kg; Sanofi Pasteur MSD).

Control rabbits received the buffer (15 mM citric acid, 75 mM NaCl, 2-hydroxylpropyl-β-cyclodextrin 5% [*w*/*w*] and 0.03% polysorbate-80 pH 6.2 [referred to as vehicle]) given either IM or IV. On average, the animals dosed with 5 × 10^9^ vp/kg of Ad26 vector received a total dose of 1.25 × 10^10^ vp (based on an average BW of 2.5 kg).

All vaccines (of clinical grade) were administered in a 0.1 mL/kg BW volume either IM (in the biceps femoris) or IV (via ear vein). Blood sampling was performed from the central ear artery. The total blood volume and sampling frequency was performed according to good ethical practices. At the end of study, rabbits were anesthetized by an IV injection of pentobarbital and euthanized by exsanguination via the inguinal blood vessels. Terminal blood sampling was performed via the inguinal blood vessels.

Mice were used to confirm observations from the rabbit study in a second species. Animals were bled prior to dosing by submandibular bleeding to obtain serum. Mice were then given a single IM immunization (50 μL/hind leg) with Ad26.COV2.S (1 × 10^10^ vp, N = 10) or Ad26.ZIKV.001 (1 × 10^10^ vp, N = 10). Twenty-four hours later, mice were exsanguinated through a cardiac puncture (serum was collected) followed by cervical dislocation under isoflurane anesthesia.

### 2.5. Droplet Digital PCR to Measure Adenovirus DNA Copies in Blood

DNA from 100 µL of blood from immunized rabbits was extracted using the DNeasy Blood and Tissue Kit (Qiagen, Louisville, KY, USA), and the isolation procedure was optimized from the original manufacturer’s instructions. DNA was eluted by performing two subsequent elution steps using 25 µL of elution buffer per step. DNA quantity and quality were assessed using the NanoDrop 2000 Spectrophotometer (Thermo Scientific, Wilmington, DE, USA).

Adenovirus DNA copies were measured with droplet digital PCR (ddPCR) in three technical replicates. In total, 5 µL of DNA per reaction was measured in a 22 µL reaction, including 10 µL 2 × ddPCR Supermix for probes (Bio-Rad, Hercules, CA, USA), 900 nM forward primer (GATAGCGGTTTGACTCACG), 900 nM reverse primer (AATGGGGCGGAGTTGTTAC), and 250 nM probe (VIC-TCCCGTTGATTTTGGTGCC-MGB), added up to 22 µL of total volume with distilled H_2_O. Samples were incubated for 10 min at room temperature before droplet generation using the Automated Droplet Generator (Bio-Rad) following the manufacturer’s instructions. PCR was performed with 40 PCR cycles (30 s at 94 °C, 30 s at 60 °C, and 30 s at 68 °C), preceded by incubation for 10 min at 94 °C and followed by incubation for 10 min at 98 °C. Droplets were read in the QX 200 Droplet Reader (Bio-Rad) and analyzed using QuantaSoft™ Analysis Pro software (version 1.0; Bio-Rad).

### 2.6. Hematology, Clinical Chemistry, Coagulation, and C-Reactive Protein (CRP) Analysis

Rabbit blood samples for hematology and coagulation were collected before treatment and at 24 h, 48 h, 72 h, 7 days, 14 days, 21 days, and 28 days post-vaccine administration. These were analyzed for hematology or coagulation parameters on an Advia2120 Hematology Analyzer (Siemens, Beersel, Belgium) and an ACL TOP500 Coagulation Analyzer (Instrumentation Laboratory, Werfen, Zaventem, Belgium), respectively. Clinical chemistry and CRP analysis were performed on serum (1.1 mL Z-gel Microtube, Sarstedt, Berchem, Belgium) collected before treatment and at 24 h (CRP only), 48 h (clinical chemistry only), 7 days, 14 days, 21 days, and 28 days post-vaccination. Clinical chemistry was analyzed on a Cobas6000 analyzer (Roche, Mannheim, Germany), while CRP was determined in serum using a rabbit CRP enzyme-linked immunosorbent assay (ELISA; Life Diagnostics Inc., West Chester, USA) on an Infinite M1000 PRO instrument (Tecan, Mechelen, Belgium) according to the manufacturer’s instructions.

### 2.7. Serum Amyloid A Analysis

Serum amyloid A was measured in mouse serum samples using the Mouse Serum Amyloid A Quantikine ELISA kit (R&D Systems, Abingdon, UK) according to the manufacturer’s instructions.

### 2.8. Histopathology

Necropsy and gross/macroscopic examinations were conducted. Histopathologic evaluation was performed in the following formalin-fixed paraffin-embedded tissues from IV dosed rabbits: macroscopically abnormal tissues, the administration site (ear vein), adrenal glands, aorta, brain, heart, intestines (colon and duodenum), kidney, liver, lung, lymph nodes (mandibular and mesenteric), mesentery with blood vessels, spleen, and stomach. Histopathology was not conducted on the IM groups in the present study, as it was already assessed in regulatory toxicity studies.

### 2.9. Detection of S Protein in Blood Using Electrochemiluminescence

Complete ethylenediaminetetraacetic acid (EDTA)-free protease inhibitor (Roche) was added to rabbit serum samples. Serum samples were centrifuged for 3 min (2000× *g* at 4 °C) to remove particulates before assay.

S-PLEX SARS-CoV-2 S detection assay (Mesoscale, Rockville, MD, USA) was used to detect S protein in the serum samples; this assay detects the presence of the S protein receptor binding domain (RBD; direct communication from the manufacturer, Rockville, MD, USA). According to the manufacturer’s instructions, phosphate-buffered saline + 0.05% Tween-20 was used as a washing buffer.

### 2.10. Statistical Analysis

Responses were log-transformed, and groups were compared using analysis of variance (ANOVA) in cases of non-censored data or a Tobit model in cases of censored data. *p* values < 0.05 were considered statistically significant. A correction for multiple comparisons was applied where indicated.

## 3. Results

To assess the potential effects of systemic Ad26.COV2.S on platelet counts, coagulation, histopathology, and acute-phase immune response parameters, rabbits received IV vaccine dose levels that were scaled to BW. Considering that the IV dosing route leads to systemic distribution of the vaccine, scaling to BW is necessary to mimic a comparable tissue exposure to that in humans. The clinical Ad26.COV2.S dose is 5 × 10^10^ vp, which translates to 1 × 10^9^ vp/kg for a 50 kg adult. This dose/kg was used as the middle dose for IV dosing in rabbits. To assess a possible dose relationship of any finding, we also tested a high dose (5 × 10^9^ vp/kg) and a low dose (0.2 × 10^9^ vp/kg). To compare the effects of IV dosing with the intended IM route of administration, the high dose of 5 × 10^9^ vp/kg was also dosed IM. The comparator adenovirus vector-based vaccine, Ad26.ZIKV.001, was administered at the high dose of 5 × 10^9^ vp/kg (IV and IM). Ad26.ZIKV.001 encodes the Zika virus M and E Env proteins [26,27]. As a reference control, the measles–mumps–rubella (MMR) vaccine was used at a dose level based on a one-year-old child scaled to BW. The rabbit study design is shown in Figure A1A.

### 3.1. IV or IM Administration of Ad26.COV2.S Was Not Associated with Changes in Hematologic, Coagulation Time, or Clinical Chemistry Parameters

The IV and IM vaccine administrations were well tolerated by all rabbits across groups. There were no unscheduled mortalities and no vaccine-related systemic clinical signs or effects on body temperature. Minimal to slight erythema at the administration site was noted in the groups (including vehicle groups) receiving an IM injection and was considered to represent the normal, expected reaction related to the IM injection procedure [27,28].

**Figure 1:**
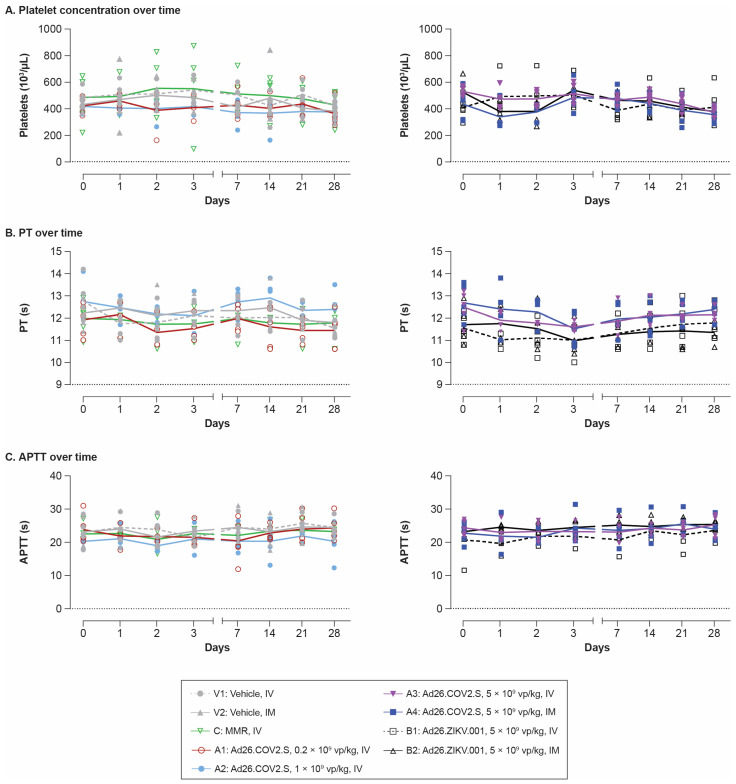
Time course of platelet, PT, and APTT levels after single IV or IM dosing with Ad26-based vaccines in rabbits. Levels of (**A**) platelet counts, (**B**) PT, and (**C**) APTT were measured in plasma taken at the indicated time points pre- and post-dosing with vehicle IV (V1); vehicle IM (V2); Ad26.COV2.S IV at 0.2 × 10^9^ vp/kg (A1), 1 × 10^9^ vp/kg (A2), or 5 × 10^9^ vp/kg (A3); Ad26.COV2.S IM at 5 × 10^9^ vp/kg (A4); Ad26.ZIKV.001 IV at 5 × 10^9^ vp/kg (B1); Ad26.ZIKV.001 IM at 5 × 10^9^ vp/kg (B2); or MMR IV (C). The time course of each parameter is shown, with lines representing the group mean and symbols corresponding to individual animals (n = 5/group) for each time point evaluated. The left graphs in each panel show the data from groups V1, V2, C, A1, and A2. The right graphs in each panel show the data from groups A3, A4, B1, and B2. APTT, activated partial thromboplastin time; IM, intramuscular; IV, intravenous; MMR, measles–mumps–rubella; PT, prothrombin time; vp, viral particles.

No clear vaccine-related changes in platelet count, prothrombin time (PT), activated partial thromboplastin time (APTT; Figure 1A,C), or any other clinical pathology parameters (except for fibrinogen and CRP, discussed in the paragraph below) were observed compared with the vehicle groups (Table A1). In addition, no major differences in these clotting parameters (platelet count, PT, and APTT) or any other clinical pathology parameters were observed between the IV and IM routes for any of the vaccines.

### 3.2. Administration of Ad26.COV2.S Led to a Transient Increase in Acute Phase Proteins

**Figure 2:**
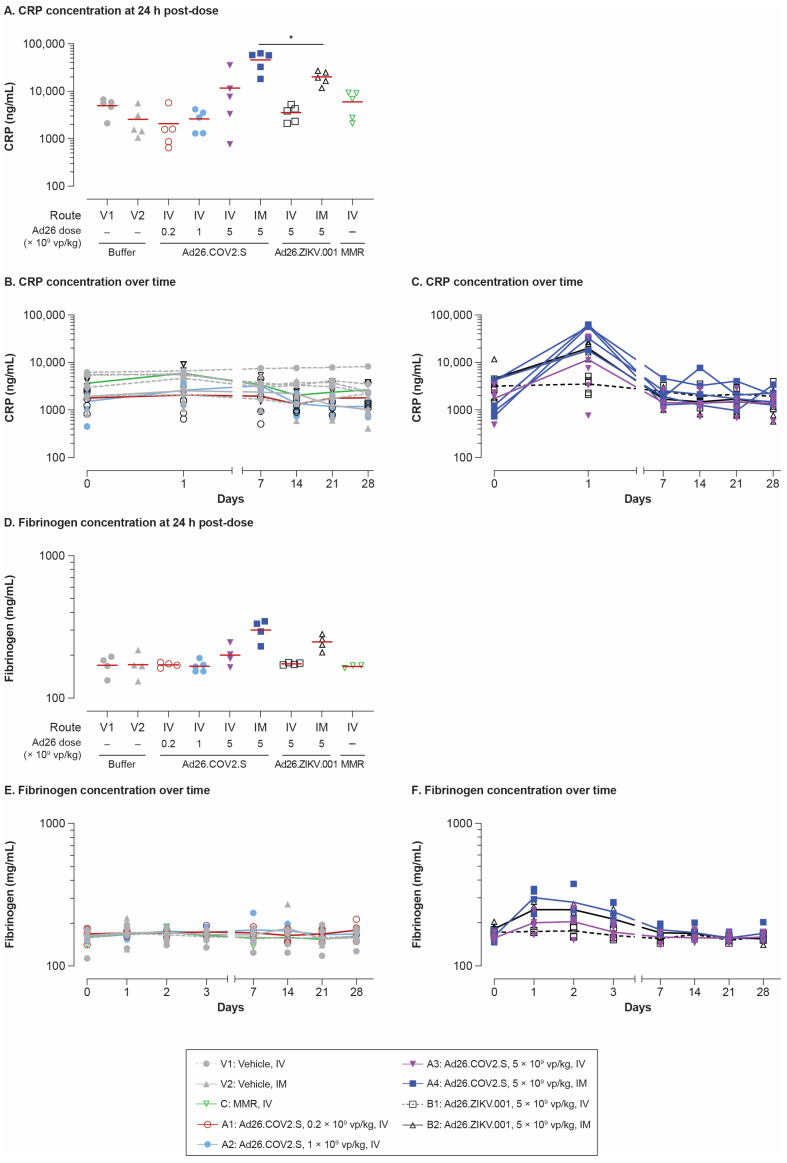
CRP and fibrinogen levels after IV and IM administration with Ad26.COV2.S and other vaccines in rabbits. Individual animal CRP (**A**–**C**) and fibrinogen (**D**,**F**) levels were measured in serum taken at the indicated time points pre- and post-dosing with the same vaccines, as indicated in the Figure 1 legend. (**A**,**D**) show CRP and fibrinogen levels at 24 h; horizontal lines represent respective group means. ANOVA testing was performed by comparing IM Ad26.COV2.S (A4) with IM Ad26.ZIKV.001 (B2). (**B**,**E**) show the time course data from groups V1, V2, C, A1, and A2, with lines representing the group mean and symbols corresponding to individual animals (n = 5/group) for each time point evaluated. (**C**,**F**) show the time course data from groups A3, A4, B1, and B2. * *p* < 0.05. ANOVA, analysis of variance; CRP, C-reactive protein; IM, intramuscular; IV, intravenous; MMR, measles–mumps–rubella; vp, viral particles.

**Figure A2:**
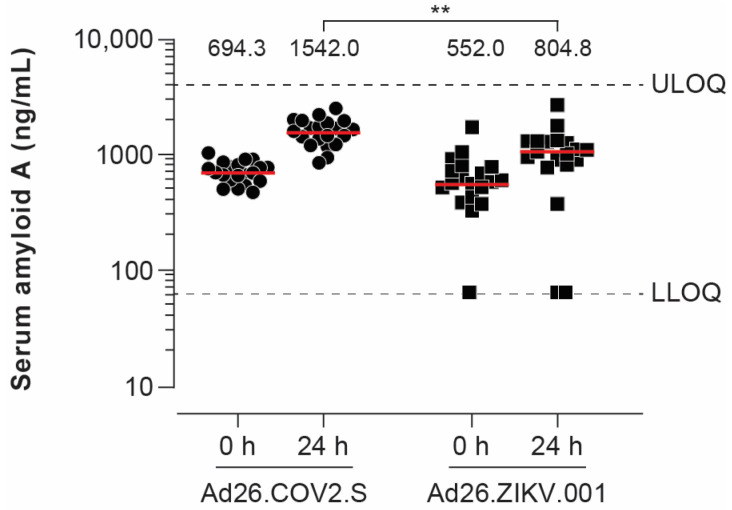
Serum amyloid A levels in mice after IM dosing with Ad26.COV2.S or Ad26.ZIKV.001. Serum amyloid A was measured in serum of mice pre-dosing and 24 h post-dosing with Ad26.COV2.S (n = 20) or Ad26.ZIKV.001 (n = 19). Each dot corresponds to an individual mouse. The numbers above refer to the geometric mean values per group; horizontal lines represent respective group means. The difference at 24 h post-dosing between Ad26.COV2.S and Ad26.ZIKV.001 was assessed (Tobit model on log_10_ transformed response). ** *p* < 0.01. IM, intramuscular; LLOQ, lower limit of quantification; ULOQ, upper limit of quantification.

Transient CRP increases (4.4- to 20.3-fold) were observed in IV and IM dosed rabbits 24 h post-dosing with 5 × 10^9^ vp/kg Ad26.COV2.S (Figure 2A–C). The CRP response was more pronounced after IM dosing, but the difference between routes was not statistically significant. There was no statistically significant difference in the CRP levels induced by IV Ad26.COV2.S (5 *×* 10^9^ vp/kg) compared with the IV reference vaccine MMR. One-week post-dosing, CRP levels in all groups had returned to baseline. Overall, the CRP increases due to Ad26 vaccination were mirrored by transient increases in fibrinogen (≤1.9-fold) observed 24 to 72 h post-dosing (Figure 2D,E). Interestingly, IM administration of Ad26.COV2.S induced significantly higher CRP levels compared with IM administration of Ad26.ZIKV.001 (*p* < 0.03, ANOVA), suggesting that the vaccine transgene influences this parameter. This observation was confirmed in a separate mouse study (the study design is shown in Figure A1B), where serum amyloid A protein levels (a mouse major acute phase protein) were significantly higher 24 h post-dosing with 1 *×* 10^10^ vp/mouse Ad26.COV2.S compared with 1 *×* 10^10^ vp/mouse Ad26.ZIKV.001 (*p* < 0.0035, Tobit model; Figure A2).

### 3.3. IV Administration of Ad26.COV2.S and Vehicle Control Induced Comparable Histopathology Findings in Rabbits

To study potential pathologic effects associated with IV dosing of Ad26.COV2.S, necropsy and gross/microscopic examinations were conducted on all IV dosed rabbits.

**Figure A3:**
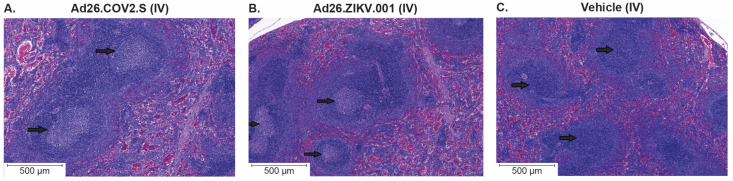
Increased cellularity and size of germinal center cellularity in spleen upon IV dosing with Ad26.COV2.S and Ad26.ZIKV.001, but not with vehicle. Representative H&E staining of spleens from New Zealand white rabbits (N = 5/group) dosed IV with (**A**) Ad26.COV2.S 5 *×* 10^9^ vp/kg, (**B**) Ad26.ZIKV.001 5 *×* 10^9^ vp/kg, or (**C**) vehicle. Arrows indicate the germinal centers. H&E, hematoxylin and eosin; IV, intravenous; vp, viral particles.

IV dosing with Ad26.COV2.S, Ad26.ZIKV.001, or the MMR vaccine was not associated with any gross or histopathologic evidence of thrombosis, thromboembolic disease, or their sequelae, as assessed following necropsy on day 28 post-immunization in comparison with vehicle controls. A comparable minimally or mildly increased cellularity of germinal centers in the spleen was observed in animals dosed IV with Ad26.COV2.S and Ad26.ZIKV.001, which is part of the normal immune response to vaccine administration (Figure A3) [28,29]. There were no systemic pathologic findings associated with IV Ad26.COV2.S or Ad26.ZIKV.001 administration. Locally, at the IV administration site (ear vein), minimal or mild, procedure-related perivenous hemorrhage, inflammatory infiltrates/inflammation, or fibrosis were observed for all IV dosed vaccines at incidence rates comparable to the vehicle controls.

### 3.4. Ad26 DNA Copies Detected in the Blood 30 Min after IV and IM Administration of Ad26.COV2.S or Ad26.ZIKV.001 in Rabbits

**Figure A4:**
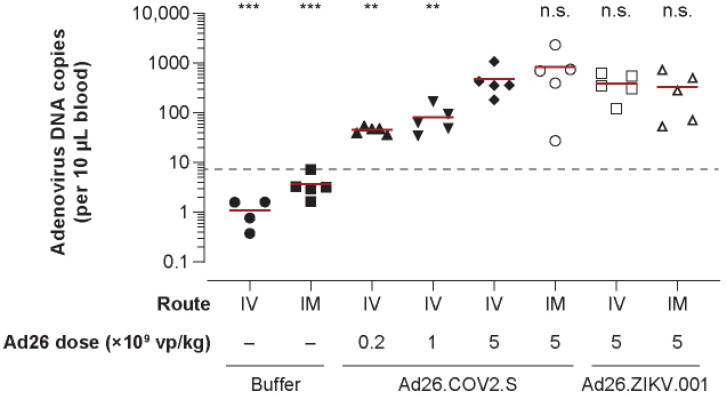
Adenovirus DNA copies in rabbit blood upon immunization. Each dot represents DNA copies/10 µL blood from an individual rabbit, of which the presence of vector DNA is measured in three technical replicates (n = three per rabbit). Both adenovirus vectors used in the study (Ad26.COV2.S and Ad26.ZIKV.001) contained a transgene cassette with a CMV promoter. The primer–probe set used in the ddPCR assay was designed to amplify a sequence from this CMV promoter. For each group (n = five rabbits per group, except for the IV buffer control group, where four samples were available), the difference with the group dosed IV with 5 *×* 10^9^ vp/kg of Ad26.COV2.S was assessed (ANOVA on log_10_ [average per rabbit] with separate variance per group). Each line represents the arithmetic mean of the DNA copies per rabbit in each group; horizontal lines represent respective group means. Threshold (dotted line) is defined as the highest value of the negative control. ** *p* < 0.01. *** *p* < 0.001. Ad26, adenovirus type 26; ANOVA, analysis of variance; CMV, cytomegalovirus; ddPCR, droplet digital polymerase chain reaction; IM, intramuscular; IV, intravenous; n.s., not significant; vp, viral particles.

To confirm IV dosing and to investigate if IM dosing resulted in distribution of vaccine components into the circulation, we quantified Ad26-derived DNA in whole blood early after administration. Ad26 vector DNA copies in blood drawn 30 min post-IV Ad26.COV2.S administration were detected at higher levels with increasing doses (0.2 *×* 10^9^, 1 *×* 10^9^, and 5 *×* 10^9^ vp/kg for groups A1, A2, and A3, respectively). Moreover, a similar number of Ad26 vector DNA copies was detected in the blood after administration with Ad26.COV2.S (IM) or Ad26.ZIKV.001 (IM and IV) at a dose of 5 *×* 10^9^ vp/kg (Figure A4).

### 3.5. IV and IM Administration of Ad26.COV2.S Vector Induced Detectable Levels of S Protein in Rabbit Serum

Considering the hypothesis of a potential role of the SARS-CoV-2 S protein (fragments) in VITT [20,21], we assessed the level of S protein in the serum of rabbits immunized with Ad26.COV2.S using a commercial S-PLEX SARS-CoV-2 S protein detection assay based on an electrochemiluminescence readout that detects the presence of the S protein RBD.

**Figure 3:**
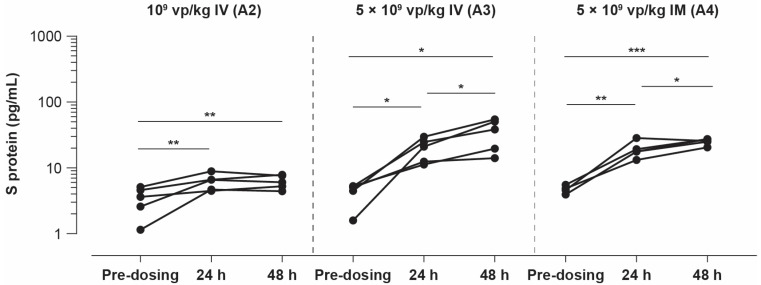
S protein concentration in serum post-dosing with Ad26.COV2.S vector encoding S protein. Sera from New Zealand white rabbits (n = 5/group) dosed with Ad26.COV2.S 1 *×* 10^9^ vp/kg IV (A2), 5 *×* 10^9^ vp/kg IV (A3), or 5 *×* 10^9^ vp/kg IM (A4) were analyzed using a commercial S-PLEX SARS-CoV-2 S protein detection assay. Serum was sampled pre-dosing and on day 1 (24 h) and day 2 (48 h) post-dosing. Comparison of the S protein concentration measured at day 1 and day 2 was performed using an ANOVA model, with a significance level of 0.05. Since the SARS-CoV-2 S protein detection assay is developed, but not qualified to test rabbit serum samples, no lower limit of detection is available for this assay. * *p* < 0.05, ** *p* < 0.004, *** *p* < 0.0001. ANOVA, analysis of variance; IM, intramuscular; IV, intravenous; S, spike; SARS-CoV-2, severe acute respiratory syndrome coronavirus-2; vp, viral particles.

Sera from rabbits dosed with 5 *×* 10^9^ vp/kg of Ad26.COV2.S showed significantly increased S protein concentrations at 24 and 48 h after IV dosing and IM injection compared with the baseline. After IV dosing, the geometric mean was 18.4 ± 1.53 pg/mL at 24 h and 31.1 ± 1.82 pg/mL at 48 h (both *p* < 0.05; ANOVA). After IM injection, the geometric mean was 18.6 ± 1.32 pg/mL at 24 h (*p* < 0.004; ANOVA) and 25 ± 1.12 pg/mL at 48 h (*p* < 0.0001; ANOVA). No statistically significant difference was observed between the IV and IM routes of vaccine administration (ANOVA; Figure 3).

## 4. Discussion

One hypothesis that was put forward to explain VITT observed with COVID-19 vaccines is unintended systemic exposure to vaccine particles resulting following their interaction with platelets, thereby inducing thromboembolic events [3,20,21,22].

Our data demonstrate that systemic exposure following IV administration of Ad26.COV2.S had no relevant impact on hematologic and coagulation parameters, including platelet counts or PT or APTT clotting times compared to the vehicle control in rabbits. IV dosing of Ad26.COV2.S and Ad26.ZIKV.001 resulted in an increased cellularity of germinal centers in the spleen, which is, however, part of a normal immune response to the injection of a vaccine [29,30,31]. Our data contrast with those published by Nicolai and colleagues [20] showing that IV injection with ChAdOx1 nCoV-19 led to a decrease in platelet count when compared with IM dosing in mice. This effect was most pronounced at dose levels above 2.5 *×* 10^8^ vp/mouse [20]. Similarly, previous studies in rabbits [32] and non-human primates [33] with high doses of Ad5 vectors encoding LacZ β-galactosidase showed a decrease in platelet count upon IV dosing with Ad5. A possible explanation for the difference between these studies and our findings is the systemic exposure dose, which requires adequate scaling between test species for the IV dosing route. In the above studies, systemic dose levels of ≥1.25 *×* 10^10^ vp/kg (mice, 20 g), 5 *×* 10^11^ vp/kg (rabbits, 2–3 kg), or 1 *×* 10^12^ vp/kg (non-human primates, 2.6–3.5 kg) were used when scaled to BW. In contrast, we tested a dose level range of 0.2 *×* 10^9^ vp/kg to 5 *×* 10^9^ vp/kg, which was selected based on the assumption that the FHD of 5 *×* 10^10^ vp Ad26.COV2.S is given to a 50 kg adult (corresponding to 1 *×* 10^9^ vp/kg) and a dose of 2.5 *×* 10^10^ vp is used for a 5 kg child (corresponding to 5 *×* 10^9^ vp/kg). We consider doses scaled to BW as more relevant for assessing IV toxicity in rabbits given the systemic exposure associated with this route compared with the local exposure associated with the IM route. Scaling to BW corresponds to a worst-case scenario where an FHD of Ad26.COV2.S is accidentally dosed IV. Of note, no indication for (pro)thrombotic events was observed in regulatory toxicology studies with Ad26.COV2.S, in which IM administration of an FHD (5 *×* 10^10^ vp) was assessed.

A second explanation for the discrepancy between our findings and those published with other adenovirus vectors might be related to the highly disparate biologic mechanisms of cell entry, receptor binding, and cell or receptor tropism used by the different vectors, as well as differences in the electronegative surface charge and vector-backbone characteristics [22,34]. These differences could potentially influence the interaction of different adenovirus-based vectors with platelets in vivo. Interestingly, a recent study showed that IV bolus injection with 10 *×* 10^11^ vp (~5 *×* 10^13^ vp/kg) of replication-competent Ad26 vectors encoding a fusion protein of green fluorescent protein and luciferase protein in human-CD46 transgenic mice failed to provoke notable changes in platelets when compared with controls [35], suggesting that even high-dose systemic exposure to Ad26-based vectors relative to BW may be tolerable, which is in line with the results of our studies.

A third explanation for the discrepancy between our findings and those published with other adenovirus vectors in the context of thrombocytopenia/VITT could be related to the levels of impurities in the vaccines. Michalik and colleagues recently showed that the Ad26.COV2.S vaccine contains much lower amounts of impurities, e.g., host cell protein, compared with ChAdOx1 nCoV-19 [36]. Additionally, no EDTA is present in the Ad26.COV2.S vaccine preparation [36], while ChAdOx1-S contains EDTA [37], which is known to activate platelets and may lead to PF4 release [38].

In our rabbit study, we observed a transient increase in acute phase proteins (CRP and fibrinogen) 24 h post-dosing, which is considered a normal response after vaccine administration, and this was more pronounced after IM immunization with Ad26.COV2.S compared with IV administration for both Ad26.COV2.S and Ad26.ZIKV.001 at a dose level of 5 *×* 10^9^ vp/kg. A potential explanation for this observation is that the IV route leads to a faster dilution of the vaccine formulation in blood compared with local injection into muscle tissue, leading to exposure of target cells to higher concentrations of the vaccine after IM administration. In addition, the procedure of IM administration causes a local inflammatory reaction at the injection site, which contributes to the change in acute phase proteins. These data are not suggestive for the use of the IV route for Ad26-based vaccines, as that would require a thorough clinical safety, immunogenicity, and efficacy assessment, which has not been conducted for IV administration of Ad26.

IM dosing of Ad26.COV2.S induced significantly higher levels of CRP (rabbits) and of serum amyloid A (mice) compared with IM dosing of Ad26.ZIKV.001, despite the fact that the adenovirus particle structure and composition is similar for both vaccines. The only difference is the genetically encoded vaccine transgene, suggesting a more inflammatory effect of the S protein encoded by Ad26.COV2.S compared with the Zika Env protein encoded by Ad26.ZIKV.001. This is supported by recent publications showing that S protein can initiate an inflammatory phenotype in endothelial cells, induce leukocyte adhesion, and promote proinflammatory cytokine secretion after IV S protein administration in mice [30,39]. Furthermore, as reviewed by Trougakos and colleagues [31,40], S protein may influence prothrombotic and inflammation-related signaling and is thus hypothesized to contribute to many adverse effects of COVID vaccination.

The development of VITT-like antibodies and adenovirus-associated thrombocytopenia and thrombosis has also been reported in two individuals experiencing natural adenovirus infection who were not previously vaccinated with adenovector-based vaccine, but who either had a prior SARS-CoV-2 infection or had received two doses of Spikvax (mRNA-based COVID-19 vaccine) [41]. The high prevalence of adenoviral infections, particularly in the developing world [42], and the limited incidence of only two VITT-like cases reported in the context of natural adenovirus infection underscores the possibility that multifactorial determinants, such as individual genetics or prior health status, may play a role in the development of VITT.

The presence of soluble S protein after vaccination with the BNT162b2 (BioNTech) vaccine was recently shown in plasma in humans [39,43] and in mice [40,44] and has been associated with the occurrence of myocarditis in young male patients dosed with the mRNA-based COVID-19 vaccine [43,45]. Notably, prolonged detection of S protein was seen in immunohistochemistry in humans up to day 60 after second dosing with BNT162b2 in the lymph nodes [39,43]. Moreover, after BNT162b2 dosing in mice, nanogram ranges (~100–400 ng/mL) of S protein were detected in the serum within 1 day after immunization and returned to background levels by day 7 [40,44]. In the present study, S protein was detected in blood on day 1 and day 2 after IV and IM dosing with Ad26.COV2.S at approximately the same level for both routes, and in the same range as seen for mBNT162b2, an mRNA COVID-19 vaccine in another animal model [44]. It remains to be determined whether a similar level of S protein expression is seen following Ad26.COV2.S vaccination in humans. Our data suggest that soluble S protein generated in the context of adenovectors is likely not sufficient on its own for the induction of VITT, since no adverse effect was observed in our study and since mRNA vaccines also induce detectable soluble S protein in the circulatory system without causing a similar frequency of VITT in human vaccinees [7,43,46]. Nevertheless, the exact location of S protein expression; the duration of expression; and the conformation, membrane presentation, or glycosylation of the S protein could be different between the vaccine platforms [45,47]. Therefore, S protein may not be ruled out as a potential contributing factor in a multifactorial scenario of VITT pathogenesis that may also include other risk factors, such as previous infections, genetic predispositions, or preexisting health conditions. Future studies need to characterize the S protein detected in the circulation in greater depth, including clarification of the S protein biodistribution in tissues other than blood and its potential influence on inflammatory processes, and comparison between different COVID-19 vaccines to assess a potential role in VITT.

## 5. Conclusions

In conclusion, we have shown that Ad26.COV2.S, independently of the administration route, did not have a relevant impact on platelet counts and other blood parameters, such as coagulation times and clinical chemistry parameters, in rabbits. Moreover, IV and IM dosing did not induce any major changes in safety parameters compared with vehicle controls or the childhood MMR vaccine when administered IV. Although based on a limited number of animals, these data indicate that an accidental IV injection of Ad26.COV2.S by itself is unlikely to represent a direct risk that could be associated with VITT pathogenesis. The very low incidence of VITT in humans suggests that this clinical outcome may be associated with Ad26 vector-related factors in combination with other factors, potentially including inflammatory activity of the S transgene and a predisposition of the host.

## Data Availability

The data presented in this study are available upon request to the corresponding author.

## Appendix A

**Table A1:** Additional rabbit hematology and clinical chemistry data after IV and IM dosing with Ad26.COV2.S, Ad26.ZIKV.001, and MMR.

Time Point	Group	Red Blood Cell Count	Hematocrit	Hemoglobin	Mean Cell Volume	Reticulocytes	Reticulocytes	Mean Cell Hemoglobin	Mean Cell Hemoglobin Concentration	White Blood Cells	Neutrophils	Lymphocytes	Monocytes	Eosinophils	Basophils	Large Unstained Cells
		10^6^/µL	%	g/dL	fl	%	10^9^/L	pg	g/dL	10^3^/µL	10^3^/µL	10^3^/µL	10^3^/µL	10^3^/µL	10^3^/µL	10^3^/µL
**Day** **−8 (pre-treatment)**	**Vehicle, IV**	5.97 (0.096)	40.5 (0.76)	12.9 (0.27)	67.9 (0.91)	1.6 (0.20)	96.8 (12.02)	21.6 (0.29)	31.9 (0.13)	7.01 (0.419)	0.93 (0.161)	5.52 (0.503)	0.12 (0.026)	0.08 (0.017)	0.34 (0.031)	0.03 (0.009)
	**Vehicle, IM**	6.20 (0.166)	39.6 (0.97)	12.7 (0.29)	64.0 (0.74)	1.3 (0.18)	80.6 (12.91)	20.5 (0.17)	32.1 (0.20)	7.84 (0.443)	0.98 (0.108)	6.21 (0.309)	0.08 (0.012)	0.10 (0.019)	0.44 (0.062)	0.02 (0.004)
	**Ad26.COV2.S 0.2 × 10^9^ vp/kg, IV**	6.07 (0.155)	39.4 (0.86)	12.8 (0.28)	65.1 (1.66)	1.9 (0.32)	111.0 (17.21)	21.2 (0.53)	32.6 (0.11)	7.70 (0.814)	1.01 (0.166)	6.09 (0.712)	0.11 (0.027)	0.09 (0.015)	0.38 (0.066)	0.03 (0.012)
	**Ad26.COV2.S 1 × 10^9^ vp/kg, IV**	6.17 (0.190)	40.7 (1.23)	13.1 (0.33)	66.1 (1.36)	1.8 (0.16)	107.3 (8.05)	21.3 (0.48)	32.3 (0.24)	6.63 (0.312)	0.90 (0.078)	5.11 (0.309)	0.11 (0.015)	0.10 (0.012)	0.38 (0.014)	0.03 (0.010)
	**Ad26.COV2.S 5 × 10^9^ vp/kg, IV**	6.04 (0.068)	39.2 (0.50)	12.8 (0.19)	65.0 (0.90)	1.8 (0.29)	110.0 (17.32)	21.3 (0.22)	32.8 (0.28)	7.40 (0.406)	1.14 (0.049)	5.70 (0.353)	0.11 (0.024)	0.08 (0.007)	0.35 (0.044)	0.02 (0.005)
	**Ad26.COV2.S 5 × 10^9^ vp/kg, IM**	6.30 (0.122)	41.9 (0.67)	13.5 (0.12)	66.6 (1.88)	1.5 (0.08)	93.0 (4.10)	21.5 (0.50)	32.3 (0.29)	8.36 (0.397)	1.20 (0.039)	6.21 (0.383)	0.15 (0.038)	0.11 (0.025)	0.46 (0.035)	0.03 (0.010)
	**Ad26.ZIKV.001 5 × 10^9^ vp/kg, IV**	6.60 (0.073)	41.8 (1.11)	13.3 (0.23)	63.2 (1.11)	2.0 (0.29)	131.6 (18.94)	20.2 (0.24)	31.9 (0.37)	6.81 (0.867)	1.10 (0.209)	5.13 (0.651)	0.11 (0.014)	0.08 (0.010)	0.37 (0.081)	0.02 (0.002)
	**Ad26.ZIKV.001 5 × 10^9^ vp/kg, IM**	6.51 (0.092)	41.3 (0.71)	13.3 (0.07)	63.6 (1.04)	1.7 (0.24)	112.2 (14.49)	20.5 (0.20)	32.2 (0.42)	8.48 (0.626)	1.29 (0.164)	6.28 (0.409)	0.23 (0.059)	0.09 (0.012)	0.56 (0.060)	0.04 (0.016)
	**MMR, IV**	6.75 (0.261)	42.9 (1.42)	13.7 (0.36)	63.6 (0.88)	1.5 (0.13)	103.0 (6.35)	20.4 (0.30)	32.0 (0.29)	7.53 (0.615)	1.11 (0.084)	5.74 (0.584)	0.24 (0.058)	0.08 (0.012)	0.32 (0.054)	0.03 (0.009)
**24 h post dosing**	**Vehicle, IV**	5.69 (0.071)	38.2 (0.40)	12.1 (0.12)	67.1 (0.25)	2.9 (0.24)	165.1 (13.36)	21.3 (0.24)	31.7 (0.28)	6.84 (0.306)	0.97 (0.066)	5.33 (0.380)	0.14 (0.043)	0.06 (0.003)	0.31 (0.011)	0.02 (0.003)
	**Vehicle, IM**	6.10 (0.260)	39.0 (1.46)	12.4 (0.48)	64.0 (0.77)	3.2 (0.27)	192.9 (15.78)	20.3 (0.24)	31.7 (0.18)	8.84 (0.402)	1.08 (0.118)	6.80 (0.173)	0.20 (0.068)	0.11 (0.020)	0.46 (0.084)	0.02 (0.005)
	**Ad26.COV2.S 0.2 × 10^9^ vp/kg, IV**	5.79 (0.273)	37.3 (0.80)	11.9 (0.30)	64.7 (1.85)	3.7 (0.28)	210.4 (7.31)	20.7 (0.68)	32.0 (0.26)	9.59 (0.728)	1.06 (0.102)	7.62 (0.598)	0.20 (0.049)	0.08 (0.008)	0.61 (0.094)	0.03 (0.009)
	**Ad26.COV2.S 1 × 10^9^ vp/kg, IV**	5.93 (0.092)	38.4 (0.62)	12.5 (0.18)	64.8 (1.44)	3.2 (0.17)	190.7 (9.49)	21.2 (0.51)	32.7 (0.20)	6.75 (0.633)	0.91 (0.106)	5.18 (0.554)	0.15 (0.025)	0.09 (0.012)	0.39 (0.032)	0.01 (0.002)
	**Ad26.COV2.S 5 × 10^9^ vp/kg, IV**	5.75 (0.062)	37.4 (0.76)	12.3 (0.21)	65.1 (1.08)	3.6 (0.14)	206.7 (9.15)	21.4 (0.31)	32.8 (0.15)	7.31 (0.579)	1.04 (0.064)	5.66 (0.431)	0.24 (0.081)	0.04 (0.008)	0.29 (0.051)	0.03 (0.011)
	**Ad26.COV2.S 5 × 10^9^ vp/kg, IM**	5.72 (0.129)	37.8 (0.38)	12.3 (0.15)	66.2 (1.82)	2.6 (0.09)	149.8 (6.52)	21.6 (0.64)	32.5 (0.12)	6.37 (0.465)	1.66 (0.138)	4.25 (0.516)	0.10 (0.036)	0.04 (0.017)	0.30 (0.059)	0.03 (0.004)
	**Ad26.ZIKV.001 5 × 10^9^ vp/kg, IV**	6.09 (0.045)	38.2 (0.73)	12.4 (0.19)	62.7 (1.01)	3.3 (0.07)	203.7 (5.83)	20.3 (0.21)	32.4 (0.24)	7.15 (0.595)	0.97 (0.117)	5.45 (0.459)	0.23 (0.039)	0.07 (0.013)	0.39 (0.064)	0.04 (0.015)
	**Ad26.ZIKV.001 5 × 10^9^ vp/kg, IM**	5.99 (0.164)	37.8 (0.76)	12.3 (0.31)	63.2 (1.00)	2.8 (0.32)	163.4 (15.63)	20.6 (0.24)	32.6 (0.34)	6.93 (0.403)	1.38 (0.201)	4.90 (0.359)	0.21 (0.028)	0.05 (0.011)	0.36 (0.050)	0.03 (0.004)
	**MMR, IV**	6.24 (0.105)	40.4 (0.52)	12.7 (0.27)	64.7 (1.14)	3.3 (0.17)	207.0 (12.89)	20.4 (0.43)	31.5 (0.28)	8.81 (0.699)	1.30 (0.200)	6.86 (0.488)	0.17 (0.053)	0.08 (0.007)	0.39 (0.066)	0.01 (0.003)
**48 h post dosing**	**Vehicle, IV**	5.67 (0.145)	38.2 (0.95)	12.1 (0.20)	67.3 (0.44)	3.1 (0.17)	175.4 (10.38)	21.3 (0.28)	31.7 (0.45)	6.31 (0.479)	0.96 (0.097)	4.72 (0.532)	0.24 (0.030)	0.06 (0.004)	0.30 (0.031)	0.02 (0.012)
	**Vehicle, IM**	5.86 (0.246)	37.2 (1.32)	12.1 (0.43)	63.6 (0.81)	3.2 (0.25)	185.5 (12.61)	20.6 (0.24)	32.5 (0.31)	8.29 (0.643)	1.12 (0.160)	6.34 (0.386)	0.25 (0.086)	0.09 (0.017)	0.45 (0.068)	0.03 (0.011)
	**Ad26.COV2.S 0.2 × 10^9^ vp/kg, IV**	5.76 (0.240)	37.4 (1.19)	12.2 (0.28)	65.1 (1.60)	3.6 (0.30)	207.0 (12.66)	21.2 (0.60)	32.6 (0.43)	9.36 (0.974)	1.02 (0.121)	7.54 (0.788)	0.13 (0.020)	0.11 (0.020)	0.54 (0.125)	0.03 (0.006)
	**Ad26.COV2.S 1 × 10^9^ vp/kg, IV**	5.92 (0.121)	38.5 (0.54)	12.5 (0.19)	65.2 (1.35)	3.5 (0.13)	207.2 (8.38)	21.1 (0.49)	32.4 (0.12)	8.42 (0.503)	1.16 (0.123)	6.36 (0.471)	0.22 (0.060)	0.13 (0.010)	0.51 (0.037)	0.04 (0.004)
	**Ad26.COV2.S 5 × 10^9^ vp/kg, IV**	5.62 (0.168)	37.5 (1.45)	11.9 (0.32)	66.8 (1.92)	4.1 (0.16)	230.7 (15.47)	21.3 (0.40)	31.9 (0.52)	8.44 (0.066)	0.97 (0.054)	6.75 (0.153)	0.28 (0.084)	0.08 (0.021)	0.33 (0.027)	0.03 (0.005)
	**Ad26.COV2.S 5 × 10^9^ vp/kg, IM**	5.32 (0.157)	36.0 (0.96)	11.7 (0.14)	67.8 (0.29)	2.6 (0.07)	139.3 (5.00)	22.1 (0.40)	32.5 (0.45)	7.82 (0.872)	0.77 (0.157)	6.19 (0.946)	0.35 (0.093)	0.05 (0.003)	0.42 (0.053)	0.04 (0.007)
	**Ad26.ZIKV.001 5 × 10^9^ vp/kg, IV**	5.88 (0.093)	37.1 (0.69)	11.9 (0.22)	63.1 (0.81)	3.6 (0.14)	210.1 (5.07)	20.3 (0.23)	32.2 (0.11)	7.50 (0.300)	0.87 (0.034)	5.70 (0.373)	0.40 (0.058)	0.09 (0.010)	0.42 (0.060)	0.03 (0.004)
	**Ad26.ZIKV.001 5 × 10^9^ vp/kg, IM**	5.90 (0.168)	37.1 (0.79)	12.1 (0.23)	63.0 (1.12)	2.9 (0.31)	169.6 (14.54)	20.6 (0.24)	32.6 (0.28)	8.35 (0.670)	0.73 (0.093)	6.82 (0.586)	0.26 (0.076)	0.06 (0.007)	0.43 (0.053)	0.04 (0.008)
	**MMR, IV**	5.98 (0.048)	38.2 (0.60)	12.3 (0.17)	63.9 (1.09)	3.4 (0.20)	202.8 (12.61)	20.6 (0.27)	32.2 (0.17)	8.57 (0.736)	1.40 (0.126)	6.41 (0.527)	0.26 (0.050)	0.11 (0.022)	0.35 (0.065)	0.04 (0.019)
**72 h post dosing**	**Vehicle, IV**	5.15 (0.055)	34.9 (0.46)	11.2 (0.20)	67.8 (0.26)	3.7 (0.46)	190.4 (22.50)	21.9 (0.18)	32.3 (0.19)	7.74 (0.905)	1.08 (0.203)	5.89 (0.731)	0.30 (0.032)	0.09 (0.027)	0.35 (0.068)	0.02 (0.003)
	**Vehicle, IM**	5.41 (0.081)	34.9 (0.76)	11.2 (0.24)	64.5 (0.65)	3.6 (0.26)	199.1 (11.78)	20.6 (0.30)	32.0 (0.43)	9.01 (0.673)	1.12 (0.109)	6.96 (0.403)	0.27 (0.111)	0.09 (0.010)	0.54 (0.106)	0.03 (0.008)
	**Ad26.COV2.S 0.2 × 10^9^ vp/kg, IV**	5.46 (0.333)	35.1 (1.14)	11.2 (0.34)	64.6 (2.37)	4.4 (0.35)	240.0 (14.73)	20.7 (1.06)	32.0 (0.47)	10.43 (1.088)	1.19 (0.214)	8.28 (0.883)	0.12 (0.023)	0.19 (0.044)	0.61 (0.140)	0.04 (0.022)
	**Ad26.COV2.S 1 × 10^9^ vp/kg, IV**	5.48 (0.081)	36.7 (0.56)	11.9 (0.13)	66.9 (0.66)	3.9 (0.26)	212.5 (13.23)	21.7 (0.27)	32.4 (0.21)	7.73 (0.996)	1.19 (0.189)	5.56 (0.751)	0.30 (0.111)	0.11 (0.010)	0.51 (0.042)	0.05 (0.018)
	**Ad26.COV2.S 5 × 10^9^ vp/kg, IV**	5.36 (0.088)	35.0 (0.69)	11.4 (0.13)	65.4 (0.84)	4.7 (0.24)	250.8 (14.72)	21.3 (0.33)	32.6 (0.40)	8.55 (0.475)	1.19 (0.117)	6.50 (0.427)	0.28 (0.055)	0.10 (0.010)	0.42 (0.045)	0.07 (0.034)
	**Ad26.COV2.S 5 × 10^9^ vp/kg, IM**	5.34 (0.081)	35.3 (0.69)	11.6 (0.23)	66.2 (1.35)	4.0 (0.34)	212.7 (18.92)	21.7 (0.49)	32.8 (0.14)	8.07 (0.623)	0.96 (0.130)	6.09 (0.560)	0.39 (0.090)	0.06 (0.005)	0.50 (0.027)	0.07 (0.018)
	**Ad26.ZIKV.001 5 × 10^9^ vp/kg, IV**	5.58 (0.045)	35.2 (0.87)	11.4 (0.23)	63.1 (1.10)	4.2 (0.26)	235.8 (16.10)	20.5 (0.27)	32.5 (0.13)	7.59 (0.276)	0.93 (0.092)	5.89 (0.256)	0.23 (0.054)	0.10 (0.010)	0.42 (0.052)	0.03 (0.005)
	**Ad26.ZIKV.001 5 × 10^9^ vp/kg, IM**	5.86 (0.156)	37.0 (0.68)	12.1 (0.24)	63.1 (0.97)	3.8 (0.27)	224.2 (14.18)	20.6 (0.29)	32.6 (0.09)	8.64 (0.576)	1.02 (0.103)	6.51 (0.499)	0.49 (0.049)	0.08 (0.009)	0.51 (0.023)	0.03 (0.004)
	**MMR, IV**	5.72 (0.121)	37.1 (0.77)	11.9 (0.19)	64.9 (1.39)	3.8 (0.24)	215.5 (17.46)	20.8 (0.32)	32.1 (0.28)	8.39 (0.475)	1.14 (0.099)	6.49 (0.416)	0.27 (0.031)	0.10 (0.013)	0.36 (0.055)	0.03 (0.010)
**Day 7**	**Vehicle, IV**	5.37 (0.152)	36.5 (0.79)	11.7 (0.21)	68.1 (0.51)	4.4 (0.31)	236.9 (14.12)	21.8 (0.37)	32.0 (0.35)	6.93 (0.571)	0.93 (0.101)	5.37 (0.523)	0.16 (0.039)	0.07 (0.013)	0.40 (0.050)	0.02 (0.007)
	**Vehicle, IM**	5.93 (0.260)	38.2 (1.42)	12.3 (0.43)	64.5 (0.69)	3.9 (0.32)	234.3 (21.25)	20.8 (0.27)	32.2 (0.21)	8.46 (0.314)	1.01 (0.061)	6.72 (0.193)	0.13 (0.024)	0.08 (0.020)	0.48 (0.079)	0.04 (0.015)
	**Ad26.COV2.S 0.2 × 10^9^ vp/kg, IV**	5.39 (0.217)	35.9 (0.78)	11.6 (0.22)	66.6 (1.43)	5.3 (0.39)	286.5 (22.62)	21.5 (0.58)	32.3 (0.25)	9.02 (0.868)	1.07 (0.125)	7.18 (0.719)	0.09 (0.014)	0.15 (0.016)	0.52 (0.103)	0.02 (0.004)
	**Ad26.COV2.S 1 × 10^9^ vp/kg, IV**	5.66 (0.167)	38.4 (1.26)	12.4 (0.34)	67.8 (1.14)	4.8 (0.33)	269.8 (20.00)	22.0 (0.29)	32.4 (0.20)	7.34 (0.837)	1.05 (0.086)	5.59 (0.811)	0.11 (0.029)	0.15 (0.033)	0.40 (0.048)	0.05 (0.009)
	**Ad26.COV2.S 5 × 10^9^ vp/kg, IV**	5.41 (0.119)	36.6 (0.75)	11.6 (0.14)	67.7 (1.11)	4.9 (0.09)	265.9 (6.47)	21.4 (0.37)	31.7 (0.30)	8.58 (0.203)	1.04 (0.061)	6.78 (0.152)	0.18 (0.038)	0.12 (0.016)	0.42 (0.038)	0.03 (0.012)
	**Ad26.COV2.S 5 × 10^9^ vp/kg, IM**	5.42 (0.146)	36.8 (0.71)	11.7 (0.20)	68.1 (1.49)	4.1 (0.27)	220.2 (11.93)	21.5 (0.48)	31.7 (0.09)	8.15 (0.256)	1.12 (0.104)	6.18 (0.263)	0.27 (0.068)	0.08 (0.007)	0.46 (0.028)	0.04 (0.020)
	**Ad26.ZIKV.001 5 × 10^9^ vp/kg, IV**	5.74 (0.124)	37.3 (1.26)	11.8 (0.35)	65.0 (1.55)	4.3 (0.25)	245.9 (12.53)	20.5 (0.34)	31.7 (0.27)	7.37 (0.292)	0.90 (0.031)	5.64 (0.305)	0.22 (0.026)	0.11 (0.012)	0.46 (0.056)	0.03 (0.012)
	**Ad26.ZIKV.001 5 × 10^9^ vp/kg, IM**	5.76 (0.099)	37.1 (0.57)	11.9 (0.13)	64.4 (1.15)	4.4 (0.25)	254.7 (11.37)	20.7 (0.30)	32.1 (0.28)	7.76 (0.396)	1.07 (0.254)	5.89 (0.424)	0.14 (0.037)	0.10 (0.019)	0.54 (0.027)	0.02 (0.013)
	**MMR, IV**	5.76 (0.156)	37.3 (0.84)	11.9 (0.31)	64.9 (0.99)	4.6 (0.34)	266.0 (25.48)	20.7 (0.39)	31.9 (0.18)	9.01 (0.551)	1.27 (0.083)	7.08 (0.624)	0.10 (0.010)	0.10 (0.018)	0.43 (0.045)	0.02 (0.005)
**Day 14**	**Vehicle, IV**	5.54 (0.135)	38.0 (0.70)	12.1 (0.18)	68.6 (0.49)	3.8 (0.34)	210.3 (17.81)	21.9 (0.29)	32.0 (0.24)	6.77 (0.460)	0.98 (0.071)	5.14 (0.428)	0.20 (0.068)	0.07 (0.007)	0.37 (0.025)	0.02 (0.002)
	**Vehicle, IM**	6.17 (0.354)	39.9 (2.04)	12.6 (0.67)	64.8 (0.74)	3.4 (0.21)	208.9 (16.48)	20.5 (0.26)	31.6 (0.16)	8.39 (0.771)	1.07 (0.248)	6.43 (0.512)	0.23 (0.051)	0.11 (0.012)	0.52 (0.066)	0.03 (0.013)
	**Ad26.COV2.S 0.2 × 10^9^ vp/kg, IV**	5.76 (0.247)	38.5 (1.14)	12.3 (0.33)	67.0 (1.55)	4.1 (0.25)	234.8 (12.79)	21.5 (0.53)	32.0 (0.16)	8.39 (1.024)	1.09 (0.130)	6.62 (0.895)	0.15 (0.020)	0.09 (0.013)	0.40 (0.100)	0.03 (0.015)
	**Ad26.COV2.S 1 × 10^9^ vp/kg, IV**	5.99 (0.129)	39.6 (0.62)	12.8 (0.21)	66.2 (1.52)	3.6 (0.13)	215.3 (6.14)	21.4 (0.61)	32.3 (0.29)	7.22 (0.775)	1.22 (0.108)	5.28 (0.677)	0.16 (0.038)	0.11 (0.015)	0.42 (0.067)	0.04 (0.012)
	**Ad26.COV2.S 5 × 10^9^ vp/kg, IV**	5.61 (0.123)	37.4 (0.40)	12.0 (0.14)	66.8 (0.89)	3.7 (0.23)	208.8 (11.20)	21.3 (0.37)	31.9 (0.15)	8.18 (0.329)	1.00 (0.065)	6.55 (0.333)	0.16 (0.033)	0.09 (0.014)	0.36 (0.040)	0.02 (0.005)
	**Ad26.COV2.S 5 × 10^9^ vp/kg, IM**	5.50 (0.100)	37.0 (0.37)	12.0 (0.18)	67.4 (1.41)	3.2 (0.22)	174.8 (9.74)	21.8 (0.51)	32.4 (0.28)	8.16 (0.872)	1.61 (0.369)	5.74 (0.491)	0.22 (0.067)	0.10 (0.012)	0.47 (0.049)	0.02 (0.005)
	**Ad26.ZIKV.001 5 × 10^9^ vp/kg, IV**	6.05 (0.109)	38.9 (1.19)	12.4 (0.37)	64.2 (1.19)	3.2 (0.21)	194.5 (9.77)	20.4 (0.28)	31.8 (0.31)	7.70 (0.433)	0.90 (0.037)	6.09 (0.406)	0.16 (0.033)	0.08 (0.009)	0.43 (0.049)	0.03 (0.008)
	**Ad26.ZIKV.001 5 × 10^9^ vp/kg, IM**	6.06 (0.091)	38.7 (0.20)	12.5 (0.04)	64.0 (1.05)	3.0 (0.15)	181.4 (6.39)	20.8 (0.29)	32.4 (0.24)	8.06 (0.666)	1.02 (0.111)	6.00 (0.501)	0.32 (0.066)	0.08 (0.007)	0.60 (0.061)	0.03 (0.009)
	**MMR, IV**	6.24 (0.093)	40.9 (0.69)	12.9 (0.17)	65.6 (0.83)	3.8 (0.16)	236.2 (9.48)	20.8 (0.31)	31.7 (0.27)	8.07 (0.432)	1.14 (0.068)	6.24 (0.445)	0.24 (0.042)	0.08 (0.009)	0.36 (0.045)	0.02 (0.002)
**Day 21**	**Vehicle, IV**	5.72 (0.127)	38.9 (0.72)	12.3 (0.21)	68.1 (0.41)	3.4 (0.23)	191.9 (12.05)	21.6 (0.27)	31.8 (0.25)	7.09 (0.381)	1.02 (0.059)	5.31 (0.450)	0.24 (0.052)	0.09 (0.012)	0.39 (0.031)	0.04 (0.009)
	**Vehicle, IM**	6.07 (0.220)	38.7 (1.18)	12.5 (0.34)	63.7 (0.94)	2.6 (0.36)	159.2 (25.25)	20.7 (0.39)	32.5 (0.23)	8.87 (0.512)	1.03 (0.123)	6.88 (0.432)	0.18 (0.048)	0.13 (0.014)	0.62 (0.053)	0.03 (0.010)
	**Ad26.COV2.S 0.2 × 10^9^ vp/kg, IV**	5.75 (0.192)	38.2 (0.83)	12.4 (0.22)	66.6 (1.58)	3.6 (0.27)	204.5 (13.29)	21.6 (0.57)	32.5 (0.24)	8.98 (0.984)	1.17 (0.147)	7.01 (0.880)	0.18 (0.037)	0.11 (0.017)	0.47 (0.079)	0.03 (0.009)
	**Ad26.COV2.S 1 × 10^9^ vp/kg, IV**	6.05 (0.211)	39.6 (0.52)	12.9 (0.17)	65.7 (1.67)	3.0 (0.22)	180.1 (10.94)	21.4 (0.55)	32.5 (0.24)	8.49 (0.513)	1.36 (0.117)	6.13 (0.570)	0.26 (0.048)	0.12 (0.017)	0.59 (0.044)	0.04 (0.008)
	**Ad26.COV2.S 5 × 10^9^ vp/kg, IV**	5.78 (0.084)	39.0 (0.20)	12.5 (0.04)	67.5 (1.00)	3.5 (0.11)	200.9 (6.54)	21.7 (0.34)	32.2 (0.22)	8.85 (0.558)	1.10 (0.085)	6.95 (0.468)	0.20 (0.042)	0.12 (0.017)	0.44 (0.051)	0.03 (0.010)
	**Ad26.COV2.S 5 × 10^9^ vp/kg, IM**	5.70 (0.120)	38.9 (0.27)	12.6 (0.11)	68.4 (1.27)	2.9 (0.13)	164.1 (5.59)	22.2 (0.53)	32.4 (0.25)	8.51 (0.418)	1.39 (0.170)	6.29 (0.445)	0.20 (0.040)	0.11 (0.010)	0.49 (0.015)	0.03 (0.010)
	**Ad26.ZIKV.001 5 × 10^9^ vp/kg, IV**	6.08 (0.070)	39.4 (0.93)	12.6 (0.28)	64.8 (1.34)	3.2 (0.19)	192.4 (12.01)	20.6 (0.31)	31.9 (0.42)	7.36 (0.414)	0.98 (0.079)	5.60 (0.381)	0.22 (0.045)	0.11 (0.009)	0.42 (0.026)	0.04 (0.007)
	**Ad26.ZIKV.001 5 × 10^9^ vp/kg, IM**	6.26 (0.109)	40.5 (0.19)	13.0 (0.07)	64.8 (1.12)	3.3 (0.18)	203.7 (8.30)	20.7 (0.27)	32.0 (0.24)	8.91 (0.690)	1.12 (0.050)	6.92 (0.692)	0.18 (0.046)	0.11 (0.009)	0.55 (0.043)	0.04 (0.014)
	**MMR, IV**	6.13 (0.078)	40.3 (0.65)	12.9 (0.17)	65.8 (0.86)	3.2 (0.08)	196.4 (6.57)	21.0 (0.26)	32.1 (0.24)	9.74 (0.691)	1.21 (0.095)	7.55 (0.582)	0.35 (0.053)	0.11 (0.013)	0.48 (0.059)	0.03 (0.006)
**Day 28**	**Vehicle, IV**	6.06 (0.168)	41.1 (1.09)	13.1 (0.24)	67.8 (0.42)	2.9 (0.26)	174.9 (19.94)	21.7 (0.33)	32.0 (0.40)	7.00 (0.532)	1.01 (0.038)	5.29 (0.495)	0.23 (0.041)	0.07 (0.012)	0.35 (0.016)	0.04 (0.011)
	**Vehicle, IM**	6.28 (0.117)	40.3 (1.10)	13.2 (0.30)	64.2 (1.24)	2.2 (0.33)	135.9 (21.01)	20.9 (0.31)	32.6 (0.37)	8.93 (0.989)	1.02 (0.192)	7.05 (0.709)	0.19 (0.065)	0.09 (0.013)	0.55 (0.115)	0.05 (0.018)
	**Ad26.COV2.S 0.2 × 10^9^ vp/kg, IV**	6.29 (0.300)	41.8 (1.30)	13.5 (0.34)	66.8 (1.61)	3.0 (0.23)	185.4 (9.46)	21.6 (0.61)	32.4 (0.24)	9.00 (0.989)	1.21 (0.147)	6.88 (0.850)	0.27 (0.037)	0.13 (0.009)	0.46 (0.096)	0.04 (0.016)
	**Ad26.COV2.S 1 × 10^9^ vp/kg, IV**	6.45 (0.107)	42.2 (0.82)	13.7 (0.21)	65.5 (1.64)	2.6 (0.19)	164.6 (10.15)	21.3 (0.49)	32.5 (0.16)	8.00 (0.419)	1.30 (0.158)	5.77 (0.547)	0.23 (0.028)	0.14 (0.015)	0.54 (0.022)	0.03 (0.006)
	**Ad26.COV2.S 5 × 10^9^ vp/kg, IV**	5.92 (0.082)	40.0 (0.66)	12.7 (0.18)	67.7 (0.94)	2.9 (0.14)	171.2 (8.56)	21.5 (0.26)	31.7 (0.09)	8.61 (0.379)	1.14 (0.096)	6.80 (0.370)	0.12 (0.016)	0.13 (0.024)	0.40 (0.049)	0.02 (0.007)
	**Ad26.COV2.S 5 × 10^9^ vp/kg, IM**	5.99 (0.146)	40.3 (0.58)	13.1 (0.09)	67.4 (1.34)	2.4 (0.13)	143.8 (4.16)	21.9 (0.48)	32.5 (0.26)	8.64 (0.392)	1.31 (0.176)	6.53 (0.486)	0.23 (0.050)	0.11 (0.007)	0.43 (0.032)	0.03 (0.008)
	**Ad26.ZIKV.001 5 × 10^9^ vp/kg, IV**	6.35 (0.080)	40.5 (1.15)	13.2 (0.28)	63.7 (1.20)	2.5 (0.27)	158.5 (17.51)	20.7 (0.24)	32.5 (0.41)	7.33 (0.422)	0.96 (0.073)	5.68 (0.451)	0.20 (0.059)	0.08 (0.003)	0.37 (0.016)	0.04 (0.008)
	**Ad26.ZIKV.001 5 × 10^9^ vp/kg, IM**	6.46 (0.154)	41.2 (0.44)	13.3 (0.17)	63.9 (1.26)	2.5 (0.17)	165.0 (7.84)	20.7 (0.36)	32.4 (0.17)	9.28 (1.279)	1.08 (0.187)	7.09 (0.877)	0.37 (0.127)	0.11 (0.015)	0.58 (0.079)	0.04 (0.018)
	**MMR, IV**	6.56 (0.114)	43.1 (0.69)	13.8 (0.19)	65.8 (0.98)	2.8 (0.17)	180.1 (10.23)	21.1 (0.33)	32.0 (0.09)	8.51 (1.112)	1.36 (0.093)	6.21 (0.984)	0.41 (0.057)	0.11 (0.014)	0.39 (0.074)	0.03 (0.007)

Additional rabbit hematology and clinical chemistry data from rabbit study—mean values per group (standard error).
